# Whole genome sequencing reveals novel *IGHMBP2* variant leading to unique cryptic splice‐site and Charcot‐Marie‐Tooth phenotype with early onset symptoms

**DOI:** 10.1002/mgg3.676

**Published:** 2019-04-25

**Authors:** Thomas A. Cassini, Laura Duncan, Lynette C. Rives, John H. Newman, John A. Phillips, Mary E. Koziura, Jennifer Brault, Rizwan Hamid, Joy Cogan, Undiagnosed Diseases Network, Christopher J Adams, David R Adams, Mercedes E Alejandro, Patrick Allard, Euan A Ashley, Mashid S Azamian, Carlos A Bacino, Ashok Balasubramanyam, Hayk Barseghyan, Alan H Beggs, Hugo J Bellen, Jonathan A Bernstein, David P Bick, Camille L Birch, Braden E Boone, Bret L Bostwick, Lauren C Briere, Donna M Brown, Matthew Brush, Elizabeth A Burke, Lindsay C Burrage, Katherine R Chao, Shan Chen, Gary D Clark, Cynthia M Cooper, William J Craigen, Mariska Davids, Jyoti G Dayal, Esteban C Dell'Angelica, Shweta U Dhar, Katrina M Dipple, Laurel A Donnell‐Fink, Naghmeh Dorrani, Daniel C Dorset, David D Draper, Annika M Dries, David J Eckstein, Lisa T Emrick, Christine M Eng, Cecilia Esteves, Tyra Estwick, Paul G Fisher, Trevor S Frisby, Kate Frost, William A Gahl, Valerie Gartner, Rena A Godfrey, Mitchell Goheen, Gretchen A Golas, David B Goldstein, Mary G Gordon, Sarah E Gould, Jean‐Philippe F Gourdine, Brett H Graham, Catherine A Groden, Andrea L Gropman, Mary E Hackbarth, Melissa Haendel, Neil A Hanchard, Lori H Handley, Isabel Hardee, Matthew R Herzog, Ingrid A Holm, Ellen M Howerton, Howard J Jacob, Mahim Jain, Yong‐hui Jiang, Jean M Johnston, Angela L Jones, Alanna E Koehler, David M Koeller, Isaac S Kohane, Jennefer N Kohler, Donna M Krasnewich, Elizabeth L Krieg, Joel B Krier, Jennifer E Kyle, Seema R Lalani, Lea Latham, Yvonne L Latour, C. Christopher Lau, Jozef Lazar, Brendan H Lee, Hane Lee, Paul R Lee, Shawn E Levy, Denise J Levy, Richard A Lewis, Adam P Liebendorfer, Sharyn A Lincoln, Joseph Loscalzo, Richard L Maas, Ellen F Macnamara, Calum A MacRae, Valerie V Maduro, May Christine V Malicdan, Laura A Mamounas, Teri A Manolio, Thomas C Markello, Paul Mazur, Alexandra J McCarty, Allyn McConkie‐Rosell, Alexa T McCray, Thomas O Metz, Matthew Might, Paolo M Moretti, John J Mulvihill, Jennifer L Murphy, Donna M Muzny, Michele E Nehrebecky, Stan F Nelson, J Scott Newberry, Sarah K Nicholas, Donna Novacic, Jordan S Orange, J Carl Pallais, Christina S Palmer, Jeanette C Papp, Loren M Pena, Jennifer E Posey, John H Postlethwait, Lorraine Potocki, Barbara N Pusey, Rachel B Ramoni, Lance H Rodan, Jill A Rosenfeld, Sarah Sadozai, Susan L Samson, Katherine E Schaffer, Kelly Schoch, Molly C Schroeder, Daryl A Scott, Prashant Sharma, Vandana Shashi, Edwin K Silverman, Janet S Sinsheimer, Ariane G Soldatos, Rebecca C Spillmann, Kimberly Splinter, Joan M Stoler, Nicholas Stong, Kimberly A Strong, Jennifer A Sullivan, David A Sweetser, Sara P Thomas, Cynthia J Tifft, Nathanial J Tolman, Camilo Toro, Alyssa A Tran, Tiina K Urv, Zaheer M Valivullah, Eric Vilain, Tiphanie P Vogel, Daryl M Waggott, Colleen E Wahl, Nicole M Walley, Chris A Walsh, Michael F Wangler, Patricia A Ward, Katrina M Waters, Bobbie‐Jo M Webb‐Robertson, Alec A Weech, Monte Westerfield, Matthew T Wheeler, Anastasia L Wise, Lynne A Wolfe, Elizabeth A Worthey, Shinya Yamamoto, Yaping Yang, Guoyun Yu, Jing Zhang, Patricia A Zornio

**Affiliations:** ^1^ Department of Medicine Vanderbilt University Medical Center Nashville Tennessee; ^2^ Department of Pediatrics Vanderbilt University Medical Center Nashville Tennessee

**Keywords:** Charcot‐Marie‐Tooth, *IGHMBP2*, intron, splicing, Undiagnosed Disease Network, whole exome sequencing

## Abstract

**Background:**

Rare variants (RV) in immunoglobulin mu‐binding protein 2 (*IGHMBP2*) [OMIM 600502] can cause an autosomal recessive type of Charcot‐Marie‐Tooth (CMT) disease [OMIM 616155], an inherited peripheral neuropathy. Over 40 different genes are associated with CMT, with different possible inheritance patterns.

**Methods and Results:**

An 11‐year‐old female with motor delays was found to have distal atrophy, weakness, and areflexia without bulbar or sensory findings. Her clinical evaluation was unrevealing. Whole exome sequencing (WES) revealed a maternally inherited *IGHMBP2* RV (c.1730T>C) predicted to be pathogenic, but no variant on the other allele was identified. Deletion and duplication analysis was negative. She was referred to the Undiagnosed Disease Network (UDN) for further evaluation.

Whole genome sequencing (WGS) confirmed the previously identified *IGHMBP2* RV and identified a paternally inherited non‐coding *IGHMBP2* RV. This was predicted to activate a cryptic splice site perturbing *IGHMBP2* splicing. Reverse transcriptase polymerase chain reaction (RT‐PCR) analysis was consistent with activation of the cryptic splice site. The abnormal transcript was shown to undergo nonsense‐mediated decay (NMD), resulting in halpoinsufficiency.

**Conclusion:**

This case demonstrates the deficiencies of WES and traditional molecular analyses and highlights the advantages of utilization of WGS and functional studies.

## 1 INTRODUCTION

Charcot‐Marie‐Tooth Disease (CMT) encompasses a group of inherited peripheral neuropathies that can be classified as demyelinating or axonal. Various types of CMT share common clinical features of atrophy and weakness, predominantly affecting the distal muscles, accompanied by areflexia and variable degrees of sensory involvement (Bird, 1993).

*Immunoglobulin Mu‐Binding Protein 2 (IGHMBP2)* [OMIM 600502] variants are one cause of CMT (Carss et al., 2017). *IGHMP2* is an ATP‐dependent DNA and RNA helicase that is expressed in high levels in neuronal cell bodies (Carter et al., 1995). It co‐localizes with the RNA‐processing machinery and plays a role in translation (Cottenie et al., 2014). Rare variants (RV) in *IGHMBP2* often interfere with ribosome binding or the ATP‐ase activity of the helicase, resulting in abnormal RNA processing which is thought to lead to alpha‐motor neuron degeneration (Grohmann et al., 2001)

RVs in the *IGHMBP2* gene have phenotypic heterogeneity but are generally classified have two distinct clinical phenotypes. One is spinal muscular atrophy with respiratory distress type 1 (SMARD1) [OMIM 604320] an autosomal recessive condition is characterized by severe neonatal polyneuropathy diaphragmatic weakness and respiratory failure in the first few years of life (Guenther et al., 2009). The other is CMT2S [OMIM 616155] which has a milder presentation with distal muscle atrophy, weakness with areflexia and relatively minor sensory involvement (Khan et al., 2017; Lim, Bowler, Lai, & Song, 2012; Liu et al., 2016; Noensie & Dietz, 2001). Poor genotype‐phenotype correlation has been reported between these clinical variants (Pedurupillay et al., 2016).

## 2 METHODS AND RESULTS

The study participants provided electronic informed consent as approved by the National Human Genome Research Institute Institutional Review Board under research protocol 15‐HG‐0130. The patient reported here was born at 38 weeks gestation, weighing 7 pounds 1 ounce, via normal spontaneous vaginal delivery to non‐consanguineous parents following an uncomplicated pregnancy. Her development was normal until around 3 months of age when her parents noticed inversion of her feet when they would hold her upright to bear weight. She was able to sit by 6 months and walked at 12–13 months but required ankle‐foot orthotics to assist with ambulation. By 18 months her parents were concerned about her muscle weakness and evaluation by orthopedics and neurology noted weakness in eversion, calf atrophy, and decreased lower extremity reflexes. She had MRIs of her brain and spine at 30 months that were reported as normal. At age 3 she had normal basic and metabolic lab evaluations, including plasma acylglycines, acylcarnitines, free and total carnitine, amino acids, very long chain fatty acids, and urine organic acids. She also underwent electromyography and nerve conduction studies which were consistent with a motor axonal polyneuropathy or a variant of anterior horn cell disease, with findings more severe distally. At age 4 muscle biopsy showed severe neurogenic atrophy with evidence of regeneration, which was also consistent with a polyneuropathy of motor neurons or an anterior horn cell disease. Also at age 4 she was evaluated in the ED for a possible seizure described as a staring episode with tonic posturing. She had another similar episode about 6 months later in the setting of a fever. She had an EEG which was abnormal, and she was started on Keppra and she has had no further episodes. She continued to have multiple ED visits over the next several years for falls and eventually was diagnosed with confusional migraines. At age 5 she was evaluated by pulmonology, who noted no respiratory concerns, aside from some mild difficulty with clearing secretions when ill. They obtained spirometry which was normal.

She was seen in genetics clinic prior to her visit in muscle disease clinic at age 5. The primary diagnostic considerations at that time were spinal muscular atrophy (SMA) type 3 and a variant of CMT. She underwent SMA type 3 testing which was negative. In muscle disease clinic it was noted that she was unable to stand from a sitting position or climb stairs. She has supra malleolar orthoses to assist with ambulation and used a walker intermittently because of frequent falls.

At age 9 she underwent whole exome sequencing (WES) which revealed a maternally inherited *IGHMBP2* (NM_0021180.2) variant of unknown significance (VUS) (c.1730T>C; p.Leu577Pro) in coding exon 12. This variant was previously reported to be pathogenic in a patient with two compound heterozygous missense mutations in *IGHMBP2* (*Guenther* et al.*,* *2009* *)*. Deletion and duplication analysis of this *IGHMBP2* was negative for copy number variants. WES also revealed two variants in the *CACNA1H* gene which is believed to be the cause of her seizures. She was referred to the Undiagnosed Diseases Network (UDN) for further evaluation.

**Figure 1:**
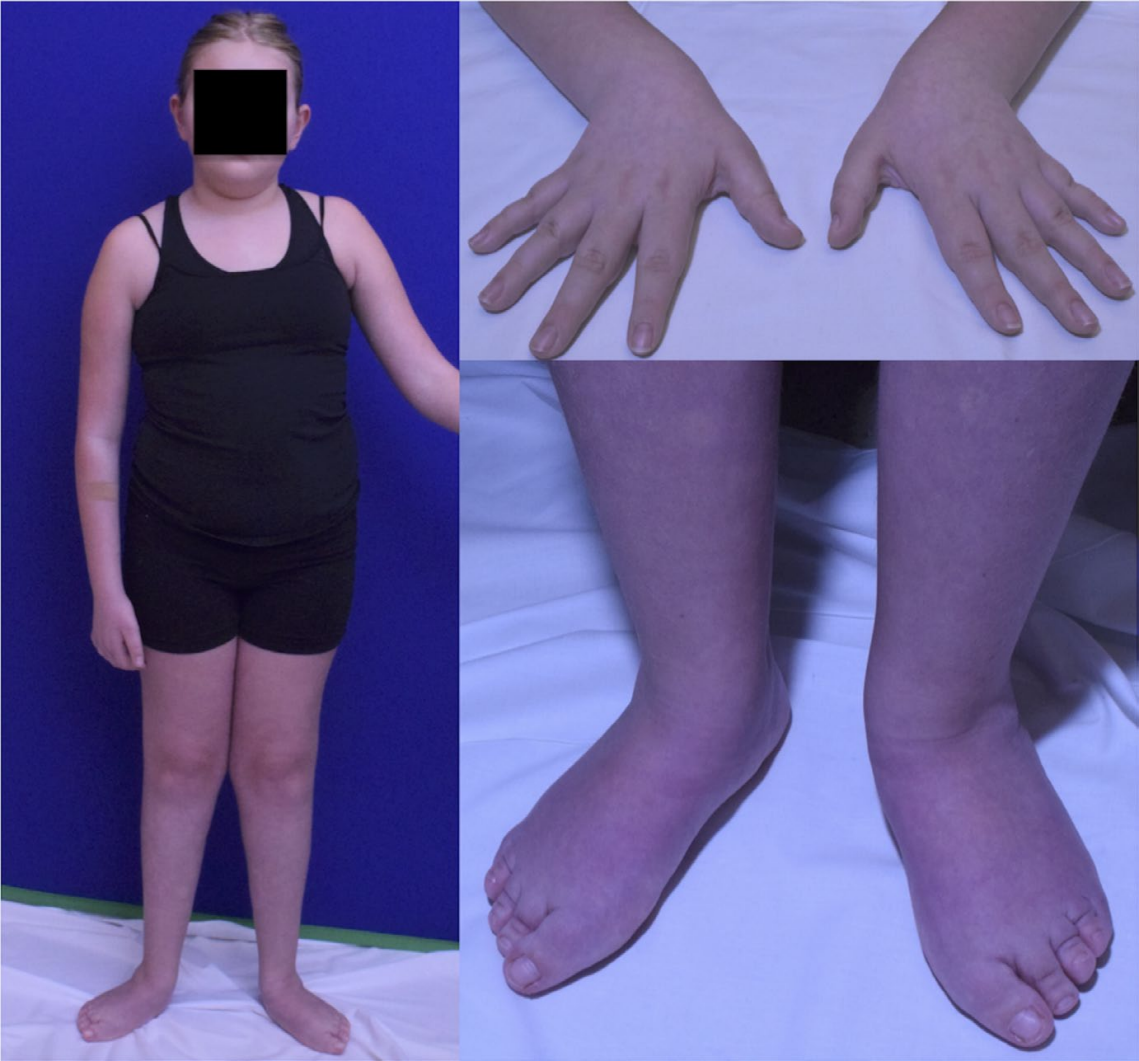
Photographs of the patient demonstrating distal atrophy

At 11 years old when she was evaluated by the UDN it was noted that she had continued difficulty with ambulation and recurrent falls. She has maintained the ability to walk over short distances. She has never been able to run or jump. She has difficulty with fine motor tasks due to weakness, but otherwise has developed typically. Her symptoms did not seem to be progressive or fatiguable. She had no bulbar or sensory symptoms. Neurologic examination showed normal mental status and cranial nerve function but symmetric diffuse weakness, more prominent distally compared to proximally, with atrophy of her distal extremities (Figure 1). Her muscle tone was normal and no fasciculations were noted. Her reflexes were absent. She had a normal sensory and coordination examination. She had a narrow based, Trandelenburg gait bilaterally with her knees locked and her feet laterally deviated. The remainder of her general physical examination was unremarkable.

Whole genome sequencing (WGS) through the UDN and data analysis using Opal software (Fabric Genomics) found the previously reported maternally inherited *IGHMBP2* missense variant, as well as an additional paternally inherited non‐coding variant (c.1235 + 894 C>A) deep in intron 8 (Table 1). The intronic variant was predicted to activate a cryptic acceptor site and was not found in any of the external databases. Both variants were Sanger confirmed.

**Table 1:** WGS identified a previously reported pathogenic variant in maternal allele and a novel deep‐intronic variant in paternal allele

Gene	Position	Change	Effect	Proband Zygosity	Mother Zygosity	Father Zygosity	1KG AF EVS AF ExAC AF gnomAD AF	SIFT Polyphen GERP		Condition
IGHMBP2	chr11: 68702864	T → C c.1730T>C p.Leu577Pro	Missense	●○	●○	○○	None	0.002		Charcot‐Marie‐Tooth disease, axonal, type 2S (AR)
								1	4.91	
IGHMBP2	chr11: 68697719	C → A c.1235+894C>A 0>8.5 Cryptic Acceptor	Intron splice site impact	●○	○○	●○	None	NA		Charcot‐Marie‐Tooth disease, axonal, type 2S (AR)

**Figure 2:**
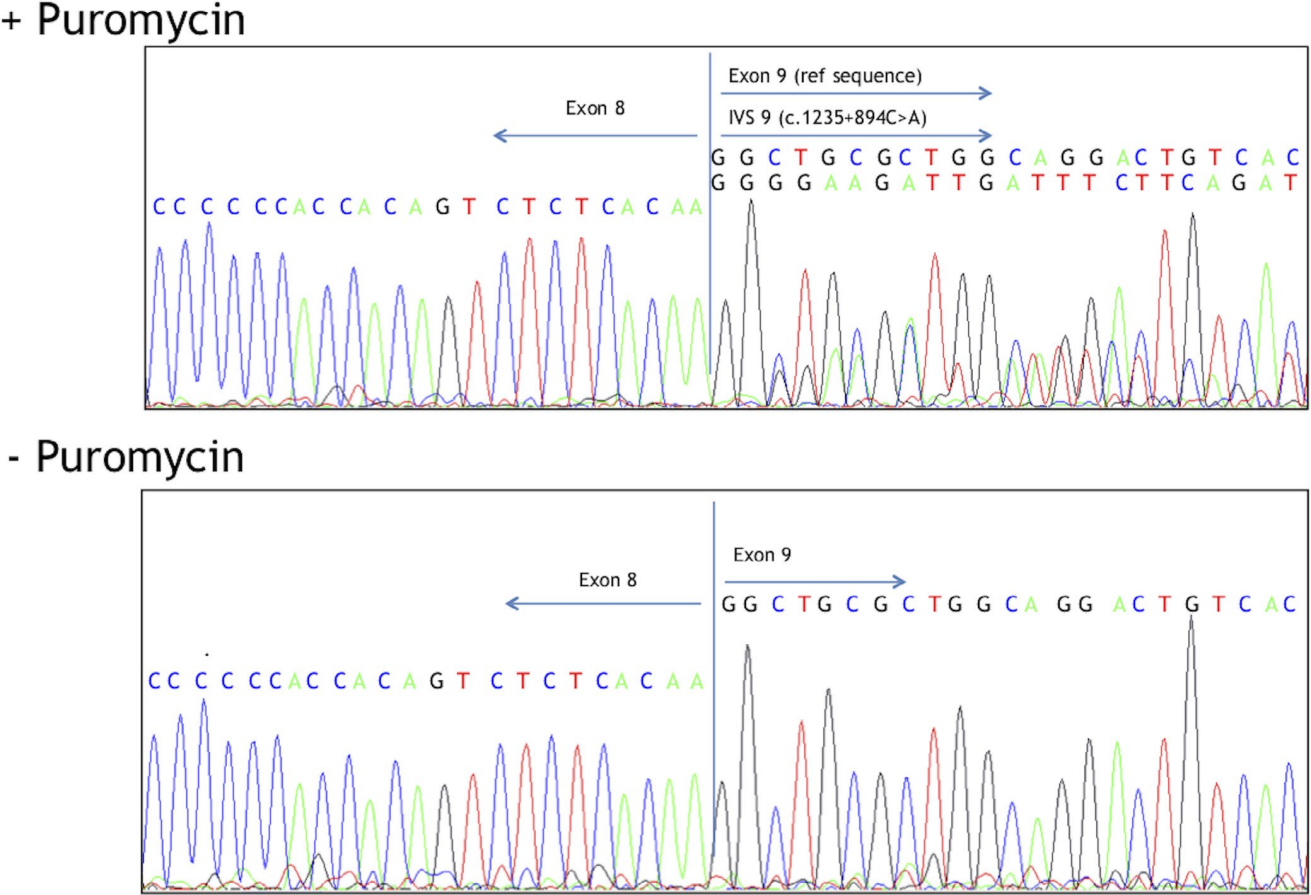
*IGHMBP2* (NM_0021180.2) cDNA sequencing in the presence and absence of puromycin, an inhibitor of nonsense ‐mediated decay (NMD). The top panel shows sequencing in the presence of puromycin demonstrating both a reference sequence of exon 9 and an intervening sequence (IVS) as a result of the c.1235 + 984 C>A variant. The bottom panel shows that in the absence of puromycin the IVS is absent, indicating it is subject to NMD

A comparison of the cryptic acceptor splice site relative to the downstream endogenous acceptor splice site, using Human Splice Finder software, predicted that the strengths of the cryptic site and the endogenous site were similar, at 91 and 87 respectively. RT‐PCR analysis of *IGHMBP2* mRNA using patient lymphocyte cells was performed with and without puromycin, which is an inhibitor of non‐sense mediated decay (NMD) (Figure 2). This revealed disruption of splicing at the site of intron 8 variant confirming that it activated the intronic cryptic acceptor site (Schottmann et al., 2015; Vanoli, Rinchetti, Porro, Parente, & Corti, 2015). Bidirectional sequencing of cDNA derived from the patient's cells identified both ends of the splicing alteration.

**Figure 3:**
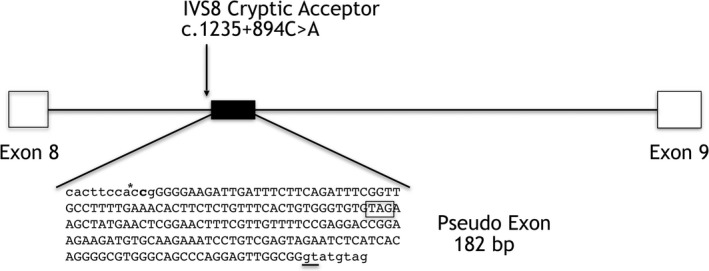
Diagram of splice site disruption of *IGHMBP2* (NM_0021180.2). Genomic localization of the pseudoexon (solid black box) with the spliced‐in 182‐bp sequence in capital letters. The location of the mutated base is indicated (asterisk), the used intronic donor splice site (GT) is underlined. The additional 182 bp results in the insertion of a stop codon (outlined boxed) and premature termination of translation

Importantly, we observed integration of a mini pseudoexon in the *IGHMBP2* cDNA that corresponded to an addition of 182 bases of intron 8 sequence. Inclusion of the 182 base pair pseudoexon causes a frameshift in the reading sequence that results in a premature stop codon (Figure 3) making the transcript subject to NMD. Absence of the transcript with the additional 182 base pair pseudoexon in the absence of puromycin indicates haploinsufficiency of the paternal allele, which leaves the only the maternally inherited *IGHMBP2* transcript that is predicted to encode a nonfunctional protein.

## 3 CONCLUSION

The current patient had a clinical presentation consistent with CMT type 2S (OMIM 616155), however thorough evaluation, including WES, revealed only a heterozygous variant in the *IGHMBP2* gene. By combining WGS data with in silico modeling and functional analyses we were able to identify a paternal deep intronic variant, which along with the previously identified maternal coding variant caused this patient's CMT2S. The difficulty of reaching a diagnosis in this patient highlights several important points.

First, despite a well characterized and distinct clinical phenotype and the availability of family members for testing, the answer was not obtained via WES. This is because WES does not capture intronic variants beyond those very close to the intron‐exon boundaries. This illustrates an important advantage of WGS in the evaluation of certain patients, because WGS can detect non‐coding sequence variants.

Second, RT‐PCR analysis using puromycin was needed to prove that the unreported non‐coding variant (c.1235 + 894 C>A) disrupted splicing and generated a premature termination codon (PTC). In addition incubation without puromycin was needed to show that this PTC resulted in activation of the NMD pathway and hapaloinsufficiency of paternal allele, which caused the patient to only have transcripts from the maternal allele which bore a previously reported pathogenic variant.

Third, accurate clinical judgement suggested the diagnosis despite negative testing. As noted above, this patient's presentation was clinically consistent with a type of CMT. Knowing that WES detected one candidate variant in the *IGHMBP2* gene which causes CMT2S allowed the UDN team to focus attention to this gene with the WGS analysis to determine if there may be an undetected non‐coding paternal variant. Even with detection of this variant by WGS, functional proof was required to determine the predicted effect on splicing. Such functional analysis is only feasible when it is guided by sound clinical judgement.

Finally, this case provides another diagnostic possibility for patient's with similar phenotypes in whom only a single potential disease causing *IGHMBP2* variant is identified. To our knowledge, our study is the first report of compound heterozygous *IGHMBP2* variants where one variant was deep intronic and affected splicing. The novel splice variant identified in this study expands the repertoire of *IGHMBP2* variants, facilitating the molecular genetic testing of future cases. This demonstrates that a second non‐coding variant missed by WES may lead to disease in patients in whom only one variant has been identified. This is true of patients who fit either phenotype, as there have been no clear genotype‐phenotype correlations, with spice site mutations being reported in both SMARD1 and CMT2S.

This case exemplifies the shifting boundary between clinical diagnoses, available clinical genetic studies (single gene sequencing, WES, WGS, and del/dup analyses), and research capabilities. While the use of WES is now accepted for diagnostic purposes our case further highlights the value of WGS and its ability to both identify novel and rare variants in coding as well as non‐coding regions. The choice between WGS or WES is not easy. WES is less expensive and more readily available, but can miss disease‐causing mutations in noncoding regions as in this case. Thus, clinicians who order WES to investigate a defined autosomal recessive phenotype should keep in mind that WES may miss a second variant non‐coding variant. WGS on the other hand captures both coding and non‐coding variants. Another additional advantage of WGS is that it has the potential to identify large structural variations such as deletions and duplications which was remains problematic with WES (Wagner et al., 2015; Yuan et al., 2017)

## CONFLICT OF INTEREST

The authors have no disclosures or conflicts of interest.
